# Mechanical ventilation in Coffin-Lowry syndrome: a case report

**DOI:** 10.5935/0103-507X.20160081

**Published:** 2016

**Authors:** Edmilson Bastos de Moura, Érica Leal Teixeira de Moura, Fábio Ferreira Amorim, Vânia Maria Oliveira

**Affiliations:** 1 Hospital de Base do Distrito Federal - Brasília (DF), Brazil.; 2 Hospital Santa Luzia - Brasília (DF), Brazil.

**Keywords:** Coffin-Lowry syndrome/diagnosis, Coffin-Lowry syndrome/therapy, Mental retardation, X-linked, Abnormalities, multiple/genetics, Case reports, Síndrome de Coffin-Lowry/diagnóstico, Síndrome de Coffin-Lowry/terapia, Retardo mental ligado ao cromossomo x, Anormalidades múltiplas/genética, Relatos de casos

## Abstract

We describe a 27-year-old patient with Coffin-Lowry syndrome with severe community pneumonia, septic shock and respiratory failure. We summarize both the mechanical ventilatory assistance and the hospitalization period in the intensive care unit.

## Abstract (pt)

Descrevemos paciente de 27 anos com síndrome de Coffin-Lowry, com quadro de pneumonia comunitária grave, choque séptico e insuficiência respiratória. Sumarizamos a assistência ventilatória mecânica, bem como o período de internação em unidade de terapia intensiva.

## INTRODUCTION

Coffin-Lowry syndrome (CLS - OMIM 303600) is a rare cause of intellectual disability that is associated with genetic inheritance linked to the X chromosome. Independently described by Coffin and Lowry, this syndrome is clinically characterized by neurological, facial, skeletal, dental and cardiac manifestations.^(1,2)^

The rarity of the condition (1:75,000 people) and the genetic heterogeneity (more than 140 different mutations have been identified in the RPS6KA3 gene, whose loss of function determines CLS)^(3)^ justify the scarcity of information on lung involvement in these individuals. Information on adult patients is also scarce due to the short life expectancy related to this condition; a literature review reports that the average survival is 20.5 years (age range of patients 13 to 34 years),^(4)^ with survival up to 39 years.^(5)^

The absence of publications with an emphasis on pulmonary performance in CLS patients with mechanical ventilation support motivated this case report. We describe a patient with CLS with respiratory failure due to community pneumonia and his need for and weaning from mechanical ventilation support during hospitalization in the intensive care unit (ICU).

## CASE REPORT

**Figure 1:**
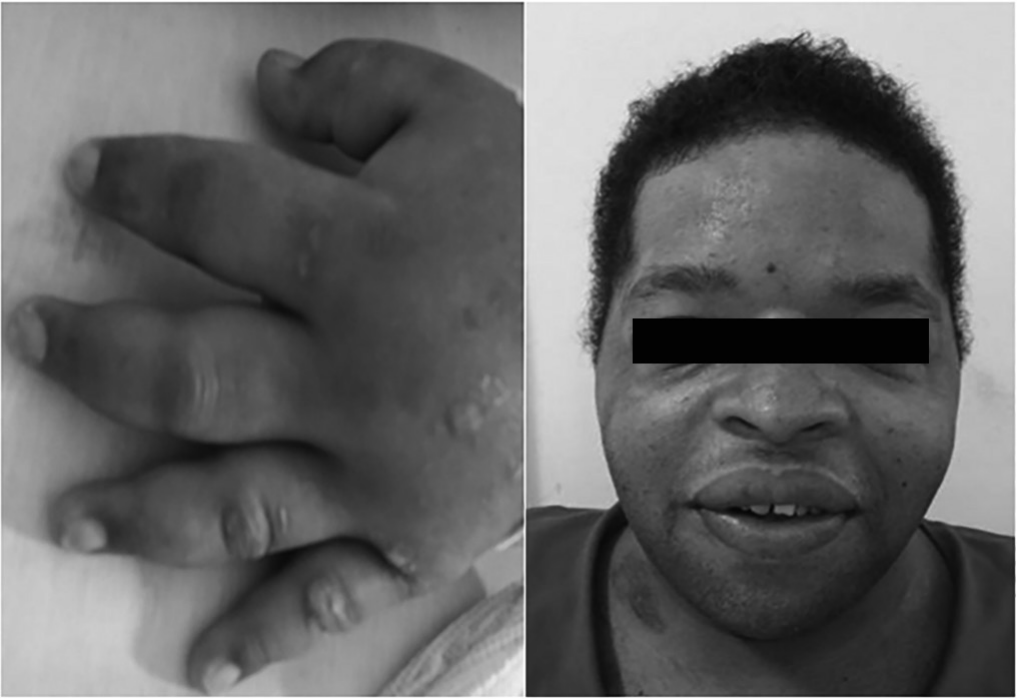
Images of the dysmorphology examination typical of Coffin-Lowry syndrome, including soft hands and wide fingers tapered at the ends and facial dysmorphism (hypertelorism, narrowing of the bitemporal diameter, down-slanted palpebral fissures, prominence of the glabella, frontal fossa, thick and everted lips, enlarged nasal base and thickened septum).

Patient ALMB was 27 years old and was admitted to the tertiary public hospital of Brasilia (DF) in June of 2015 due to progressive dyspnea, cough with purulent sputum and a fever of 38.5°C during the 10 days prior to hospitalization. The patient reported acute sinusitis in the month previous to the hospitalization and had taken amoxicillin/clavulanate for 10 days. He was treated for Hodgkin's lymphoma (nodular sclerosis type) in 2008 and met the healing criteria. The physical examination revealed the external changes and facial dysmorphism typical of CLS (Figure 1), a brevilineal body type, obesity grade II (body mass index of 37.6), and a mitral systolic murmur that was discreet and without irradiation.

**Figure 2:**
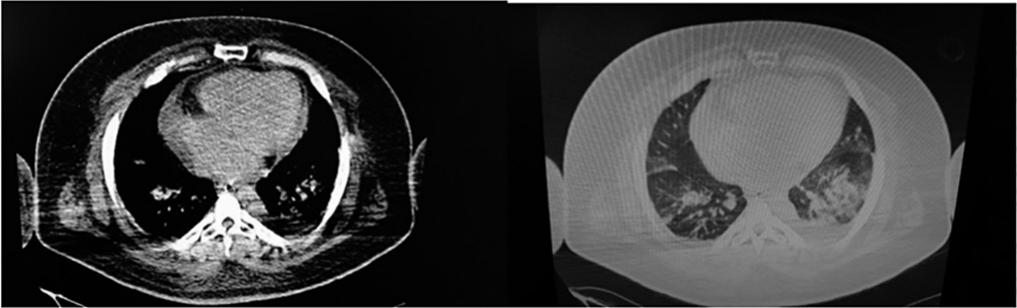
Tomographic images of the patient's chest, revealing bilateral infiltrates.

Associated with the clinical picture described above, the patient presented with polyglobulia and plaquetopenia that evolved into epistaxis during hospitalization. The chest computed tomography (CT) showed a reduction of the right lung volume with signs of ipsilateral hypoaeration and condensation with air bronchograms in the lower lobes (more extensive on the right) in addition to cardiomegaly (Figure 2). The two-dimensional transthoracic echocardiogram revealed light mitral and tricuspid insufficiency associated with light pulmonary arterial hypertension (average pulmonary arterial pressure estimated at 34mmHg).

The patient was hospitalized with a diagnosis of severe community pneumonia, and antibiotic therapy was initiated with amoxicillin/clavulanate. Due to the hematological changes, a relapse of lymphoma and idiopathic thrombocytopenic purpura were investigated but discarded based on the clinical and laboratory criteria.

The patient worsened with progression to an infectious and respiratory condition with maintenance of dyspnea and fever. The blood cultures did not show bacterial growth. An empirical antibiotic escalation to cefepime and azithromycin was performed on day 10 and to meropenem and linezolid on day 11 of hospitalization due to worsening of the clinical condition.

On day 15 of hospitalization, the patient presented with acute respiratory failure with a need for orotracheal intubation and observations of a difficult airway (Mallampati scale 4). Continuous sedoanalgesia and mechanical ventilation support were initiated to maintain the partial pressure of the oxygen/inspired fraction (PaO_2_/FiO_2_) at 168. He presented hemodynamic instability when norepinephrine was initiated and was diagnosed with septic shock and moderate acute respiratory discomfort syndrome (ARDS).

Initially, the patient was kept under assisted-controlled ventilation with pressure-cycled inspiratory pressure. The inspiratory pressure was maintained with a tidal volume between 3 and 6mL/kg of predicted weight, thereby limiting the maximum distension pressure at 15cmH_2_O and the maximum plateau pressure at 30cmH_2_O. The final positive expiratory pressure (PEEP) was adjusted according to the best complacency point (PEEP-complacency technique) with the value fixed at 12cmH_2_O. The respiratory frequency was set at 20irpm. The FiO_2_ was intended to keep the arterial oxygen saturation (SpO_2_) above 92%, and the maximum FiO_2_ used was 80%. No alveolar recruitment, neuromuscular block or prone position maneuver was performed.

Noradrenaline was used for a short period of time (48 hours) at low doses (up to 0.1µg/kg/minute). During the first six days, invasive ventilation support was maintained without difficulties with improved gas exchange and mechanical ventilation. The distension pressure was always maintained at ≤ 15cmH_2_O, and the plateau pressure was maintained at < 30cmH_2_O. In relation to the monitoring of mechanical ventilation, the static complacency of the respiratory system (Cst) was always > 30mL/cmH_2_O, and the maximum resistance of the airways (Rva) was 18cmH_2_O/L/s.

**Figure 3:**
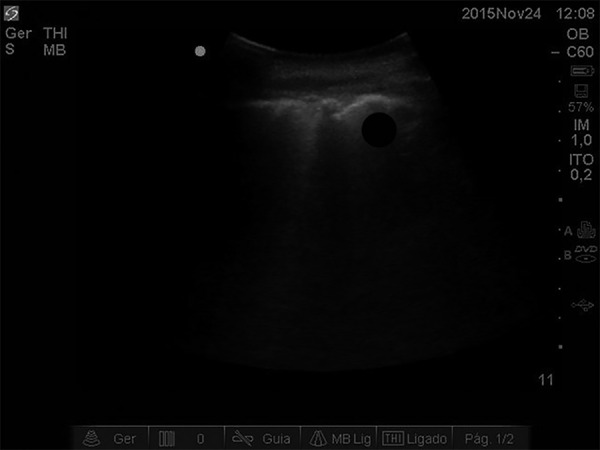
Ultrasound images of the patient's lung, revealing B lines.

On day 7 of mechanical ventilation (MV), a sudden worsening was observed, with an increase in the parameters (inspiratory pressure and FiO_2_) associated with ventilator asynchrony, increased Rva and reduced Cst. A massive amount of semi-thick secretion was observed with the formation of obstructive plugs. The chest X-ray performed on that day revealed atelectasis at the base of the right lung. After bronchial hygiene maneuvers, the clinical status was restored, and the previous parameters were achieved with improvement in the Cst and Rva values (days 8 and 9 of MV). However, on the 9^th^ day of MV, new worsening was observed in the PaO_2_/FiO_2_, with the need for high FiO_2_. Ultrasound of the lung showed B-lines (Figure 3), but a CT scan of the chest revealed no significant atelectasis areas. When the PEEP was adjusted to 12cmH_2_O and the water balance was made negative, an increment in the oxygenation index to 175 was observed within 24 hours.

With clinical improvement, ventilation weaning occurred with good evolution through the progressive reduction of pressure support (PS). Because the patient maintained a PaO_2_/FiO_2_ ratio of 280, arterial pH of 7.37, good level of consciousness based on the ability to obey four commands (open eyes, track examiner, shaking hands and protrude the tongue), hemodynamic stability, normal axillary temperature, laboratory test values and a balanced water balance, on day 15 of respiratory support the spontaneous breathing test was performed with the mechanical ventilator adjusted to the pressure support mode (PSV) with the PS adjusted to 7, a PEEP of 5cmH_2_O and FiO_2_ of 25%. After the test, the rapid shallow breathing index (or Tobin index) was 80 breaths/min/L, the systolic blood pressure was 106mmHg, the cardiac frequency was 102 beats/minute, and no change was detected in the level of consciousness. Thus, extubation was successfully performed. Due to the presence of comorbidities and obesity grade II (both considered relative criteria), the patient was submitted to preventive non-invasive ventilation with one full-face mask per day for two 2-hour periods.

The patient was discharged from the ICU after 72 hours under spontaneous breathing and remained for 6 days in the Hematology ward. Then, the patient was discharged after a 41-day hospital stay.

## DISCUSSION

The patient in question has a rare syndrome with above average survival compared with his peers, especially considering his Hodgkin's lymphoma morbid antecedent. The patient was seen by a geneticist in 2014 at the age of 25 years when the possibility of CLS was proposed. Because the patient is the only child with the disease of a non-consanguineous couple with three other male children, we believed that this case is an example of a *de novo* mutation that is very prevalent and was present in up to 68% of CLS patients in a series of cases.^(6)^

Invasive ventilation support in these patients has not been addressed in detail in the medical literature. Aspiration pneumonia may be the most prevalent cause of respiratory failure among these patients. Stimulus-induced drop episodes (SIDEs), which are present in 20% of patients, are followed by quick recovery of muscle tone without loss of consciousness^(7)^ and are not a cause of bronchoaspiration. Feeding problems, vomiting and gastroesophageal reflux have been reported,^(8)^ which when associated with neurological commitment can create a favorable scenario for bronchoaspiration. The patient in this study had a report of SIDE in February 2013 but no history of feeding difficulties. However, relatives reported a recent episode of pneumonia treated 3 months prior to admission.

Interstitial pneumonia secondary to chemotherapy with bleomycin, which was used by the patient at the time of lymphoma treatment, was an evaluated differential diagnosis. However, this hypothesis was discarded through clinical evolution and mechanical ventilation, and the chest CT was not suggestive of this diagnosis. Chest deformities were not observed, including *Pectus excavatum* or *carinatum*, which are prevalent in CLS patients. Although the clinical status was not suggestive, a histopathological examination that rejected pulmonary emphysema or pulmonary fibrosis due to chronic incipient aspiration was performed because these findings were possible in patients with this syndrome.^(9)^

The patient remained under ventilation support for 15 days. The day when support began was marked by acute hypoxemic respiratory failure. Moderate ARDS was diagnosed as proposed by the Berlin Definition (acute start, PaO_2_/FiO_2_ between 101 and 200 with PEEP ≥ 5cmH_2_O, bilateral lung condensation and respiratory failure not clearly explained by heart failure or overhead volume).^(10)^

Due to the diagnosis of moderate acute respiratory distress syndrome (ARDS), a protective ventilation strategy was adopted with the use of low tidal volumes according to the Brazilian Guidelines for Mechanical Ventilation of 2013.^(11)^ This approach consists of the use of tidal volumes of 3 to 6mL/kg of the predicted weight to maintain a maximum distension pressure of 15cmH_2_O and a plateau pressure up to 30cmH_2_O. Because several methods have been proposed to adjust the PEEP in ARDS without clear evidence of superiority, a PEEP-complacency technique was chosen in which a PEEP of 2cmH_2_O above the best complacency was established.

Because the patient maintained the PaO_2_/FiO_2_ above 150, a prone position was not adopted. Moreover, we decided not to conduct recruitment maneuvers because the distension pressure remained below 15cmH_2_O without major difficulties in tuning the mechanical ventilation. Similarly, a neuromuscular blocker was not used because the PaO_2_/FiO_2_ was greater than 120.^(11)^

With protective ventilation maintenance, progressive clinical improvement was observed during the first 6 days of MV, with a reduction of the ventilation parameters (i.e., inspiratory pressure and FiO_2_). This clinical improvement of the gaseous exchanges and mechanical ventilation were due to the action of the antibiotic therapy, the septic shock resolution and the reduction of the inflammatory process.

The instability of the ventilation status observed on day 7 of the mechanical ventilation, with increased Rva and reduced Cst, was explained by the formation of obstructive plugs and atelectasis in the lower right lobe, which are complications that must be researched in the face of acute changes in patients under MV. The worsening of gas exchanges observed on the ninth day of MV was attributed to pulmonary congestion by water excess because the changes were quickly reversed by making the water balance negative and slightly increasing the PEEP to 12cmH_2_O. The B-lines in the pulmonary ultrasound corroborated the diagnostic hypothesis of pulmonary congestion. Additionally, antibiotic escalation had already occurred to keep the infectious status under control despite the severity associated with septic shock under treatment.

The continuous administration of analgesia with opioid (fentanyl) was maintained with progressive withdrawal to reduce sympathetic stimulation and prevent the increase in peripheral vascular resistance and, consequently, myocardial stress.^(12)^ Decreasing pressure support was used until the time of extubation on the fifteenth day of support. Due to the favorable evolution, we made a choice to not perform a tracheostomy before the possibility of extubation success.

We believe that the successful ventilation weaning occurred due to the absence of cardiomyopathy in the patient. The existence of comorbidities such as cardiomyopathies associated or not with valvulopathies is common in individuals with CLS. The momentary dependency on the vasopressor corroborates the echocardiographic findings of good heart performance.

Despite mortality associated with this clinical entity (32%), the patient had a favorable evolution and remained in respiratory support during the time expected for moderate ARDS by the literature (4 - 14 days).^(10,12,13)^

We hope that similar reports will enrich our knowledge of patients with CLS under mechanical ventilation support, allow the establishment of ventilation strategies and provide specifics on the syndrome. Recognizing specific patterns of organic responses in these individuals when under critical care will result in agreed-upon actions.

## CONCLUSION

The present report described pulmonary mechanic characteristics and the clinical evolution of a patient with Coffin-Lowry syndrome stricken by severe community pneumonia and progression to septic shock with the need for mechanical ventilation support. Despite the moderate acute respiratory discomfort syndrome and severe infection, satisfactory improvement was observed during hospitalization.
